# Patient, medical and legal perspectives on reentry: the need for a low-barrier, collaborative, patient-centered approach

**DOI:** 10.1186/s40352-021-00161-7

**Published:** 2021-12-02

**Authors:** Zoe Pulitzer, Maria Box, Laura Hansen, Yordanos M. Tiruneh, Ank E. Nijhawan

**Affiliations:** 1 Department of Internal Medicine, Division of Infectious Diseases and Geographic Medicine, University of Texas Southwestern Medical Center, 5323 Harry Hines Blvd, Dallas, TX 75390 USA; 2 Department of Emergency Medicine, University of Texas Southwestern Medical Center, 5323 Harry Hines Blvd., Dallas, TX 75390 USA; 3 University of Texas Health Sciences Center at Tyler, 11937 US-271, Tyler, TX 75708-3154 USA; 4 Parkland Health and Hospital Systems, Correctional Health, Dallas, TX 75235 USA

**Keywords:** Jail, Prison, Reentry, HIV, Substance use disorder, Community health worker, Qualitative methods

## Abstract

**Background:**

Worldwide, the United States has the highest incarceration rate per capita. Thousands of people are released from US correctional facilities each year, including many who are impacted by HIV infection and substance use disorder (SUD), two frequently comorbid conditions that present multiple challenges upon reentry. Reentry and care engagement research involving justice-involved people with HIV (PWH) with comorbid SUD has been largely limited to the perspective of those released. To formulate effective interventions for this population aimed at maintaining health and reducing recidivism, it is crucial to collect data from formerly incarcerated individuals with firsthand experience of the reentry process as well as other actors within the reentry framework. Insights from medical and legal service providers working in reentry systems have the potential to address key implementation concerns. To inform an intervention aimed at helping recently-released individuals PWH and SUD, we conducted a qualitative study to assess barriers and facilitators to community reentry from the perspectives of diverse consumers and providers of medical, legal, and reentry services.

**Results:**

Fifteen stakeholders within XXX County participated in in-person interviews. Results indicated that 1) Patients/clients emphasized psychosocial support and individual attitude more than medical and legal participants, who chiefly focused on logistical factors such as finances, housing, and transportation; 2) Patients/clients expressed both medical and legal needs during the reentry period, though medical providers and participants from legal entities mainly expressed concerns limited to their respective scopes of work; 3) All three participant groups underscored the need for a low-barrier, collaborative, patient-centered approach to reentry with the goal of achieving self-sufficiency.

**Conclusions:**

Findings support and extend existing literature detailing the barriers and facilitators to successful reentry. Our findings underscore the notion that an effective reentry intervention addresses both medical and legal needs, includes an individualized approach that incorporates psychosocial needs, and focuses on establishing self-sufficiency.

## Introduction

The United States has the highest incarceration rate per capita globally, with 830 out of every 100,000 adults in prison or jail (Maruschak & Minton, 2020), many of whom are released back into their respective communities each year. HIV infection is prevalent within the incarcerated population; approximately 1.3% of those in state and federal correctional facilities have been diagnosed with the virus (Maruschak & Bronson, 2017). Comorbid substance use disorder (SUD), often associated with poor reentry health outcomes (Binswanger et al., 2007; Massoglia & Pridemore, 2015) is common among people with HIV (PWH). Nationwide, PWH have been diagnosed with at least one type of SUD and polysubstance use disorder at rates of 48% and 20%, respectively (Hartzler et al., 2017). PWH and people with SUD face multiple challenges with linking to and engaging in health care following release from incarceration. Our group found that less than 35% of PWH who returned to the community from jail were linked to HIV care within 90 days (Ammon et al., 2018). Among PWH, recidivism (Fu et al., 2013) and SUD (Wilson et al., 2011; Zgoba et al., 2020) may result in interruptions in community-based care. These concurrent legal and medical issues highlight the complex nature of reentry after incarceration for PWH and people with SUD, necessitating multidisciplinary solutions.

One solution to help mitigate complex medical needs and social determinants of health during the high-risk reentry period is the utilization of community health workers. Community health workers provide advocacy, education, encouragement, and care coordination to individuals who may need extra support navigating their medical care and engaging with social services. The benefits of using community health workers in the justice-involved population have been presented by Wang et al. (2012), whose employment of formerly incarcerated community health workers engaged individuals with chronic illness in primary care upon reentry and resulted in significantly lower rate of emergency department use. Moreover, Wang et al. (2019) highlight that working with a community health worker upon reentry was associated with lower rates of arrest for technical violations of probation and parole. The current study includes a line of questioning specific to community health workers, as we hypothesize that medical and legal service providers, as well as the people they serve, may condone the use of community health workers during the reentry period.

While prior research has focused on identifying and overcoming reentry barriers, the literature around justice-involved PWH with comorbid SUD has been largely limited to the perspective of those released. Multiple qualitative studies cite housing, transportation, substance use, stigma, social support, and employment as significant determinants of post-release engagement in HIV care (Dennis et al., 2015; Haley et al., 2014; Remien et al., 2015). Some studies have included valuable viewpoints of health care providers who work along the HIV care cascade and/or reentry process (Dong et al., 2021; Grau et al., 2017; Sidibe et al., 2015; Taylor et al., 2018), but the body of reentry literature pertaining to HIV is missing key qualitative insight from representatives of other reentry services providers, community supervision organizations, and law enforcement. People returning to the community following incarceration frequently have parole or probation requirements that pose additional barriers to HIV care, yet representatives from this field have yet to be meaningfully included in care continuity research. These individuals are uniquely positioned to provide a view of the legal services and reentry requirements after release and how they may affect health outcomes. To form and implement interventions that are effective and sustainable, it is necessary, but not sufficient, to collect data from formerly incarcerated individuals with firsthand experience of the reentry process. Including the medical and legal service providers’ perspectives can address pressing feasibility and implementation concerns, thus providing a more comprehensive overview of challenges to reentry.

To inform a community health worker intervention for recently released PWH and SUD, we conducted an initial qualitative study to assess barriers and facilitators to community reentry from the perspectives of diverse stakeholder groups: consumers, henceforth referred to as patients/clients, as well as providers of medical, legal, and reentry services.

## Methods

### Study sample

Qualitative semi-structured interviews were conducted with 15 individual stakeholders in XXX (city), XXX (state) to examine perspectives on reentry and care reengagement after incarceration as well as the utility of community health workers during the reentry period. To ensure collection of a variety of viewpoints, three different categories of stakeholders were interviewed: patients/clients, medical providers, and representatives from legal entities. Specific job titles and roles within the medical provider and legal entities groups have been withheld to prevent participant identifiability. The University of XXX Institutional Review Board approved all study procedures.

### Data collection

A purposive sample of participants was approached and recruited based on their personal experience, medical and security knowledge from these organizations: (1) XXX a XXX-based, attorney-led diversion and reentry brokerage network that collaborates with specialty courts, offers post-release legal advocacy services, and provides reentry plans and referrals to social service organizations for individuals leaving prison and jail (2) the XXX clinic, a safety net community-based clinic that provides HIV services, (3) the XXX County jail and (4) the local Sheriff’s department. Patients/clients comprised individuals who had a history of incarceration, some living with HIV and/or SUD, and previously-incarcerated individuals receiving reentry services at XXX [re-entry agency]. Medical providers included both HIV and non-HIV clinicians and nurse case managers working in jail- and community-based settings. The legal entities group was comprised of jail security staff and reentry specialists and advocates from XXX [re-entry agency].

Interview guides included the same domains for all participants, with the first half of the interview consisting of tailored questions about experiences with reentry services or the participant’s specific job function in the reentry arena. Questions about perspectives on reentry were slightly different based on the participant group; interviews with people who were formerly incarcerated included questions about firsthand experience with reentry, while the medical and legal participants discussed reentry through the lens of their jobs. The patient/client-directed questions addressed: experiences at the HIV clinic, barriers/facilitators to medication and appointment adherence, possible experiences with substance use, possible experiences during incarceration and challenges faced upon reentry, and experiences with HIV case management. The medical provider-directed questions addressed: preparing patients for release from jail and subsequent follow-up, protocol and process measures at the jail and community HIV clinic, challenges commonly faced by patients in keeping appointments and filling medications, and experiences working with patients recently released from jail/prison. The legal entity-directed questions addressed: explanation of jail culture, barriers to reliable discharge planning from jail, and challenges with post-release service provision and uptake.

The second half of the interview guide focused on a proposed community health worker intervention and research study aimed at increasing engagement in HIV and substance use care following release from incarceration and reducing recidivism. Interviewers inquired about the value of community health workers in the reentry period to assess feasibility of integrating them into post-release care and explored patient/client feelings about working with them one-on-one. Participants provided feedback on the purpose and plan, execution of the study, and brainstormed potential challenges to implementation. Interviews were administered using an open-ended method with probing as necessary. Sample interview guide questions are provided in Table 1.

**Table 1:** Sample questions from interview guides for medical providers, legal participants, and patients/clients

Topic	Medical and legal	Patient/clients
**Needs and Concerns**	What are the most common needs/concerns that are communicated by individuals who are incarcerated?	What would you say your most important need is right now?
**Perspectives on Reentry**	What are your thoughts in general about reentry and the issues surrounding recidivism?	Tell me about what it was like when you were released from jail (for the first time and so on).
**Community Health Workers**	What do you think about having a Community Health Worker help HIV+ individuals navigate care?	Do you know anything about Community Health workers (CHWs)? a. Have you ever worked with one? Where? b. If yes, how did you feel about it? What was your experience with him/her? c. If not, what ways do you think a CHW could help you?
**Setting Up for Success**	How would you characterize a successful client?	Are there any other ways that you think the release process can be improved or any other thoughts you have about inmates coming out of jail?

Individual face-to-face interviews were conducted by research personnel trained in qualitative methods, in English, in a setting that ensures the privacy of the participants. Each interview lasted between 0.5–1.5 h. The interviews took place over 3 months in one of three locations: in a conference room at XXX [re-entry agency], in a patient room at the XXX HIV clinic, or in a conference room in the XXX County jail. All participants were provided with a study information sheet and participants provided verbal consent. Each interview was audio-recorded with a handheld voice recorder and transcribed by a professional transcription service. At the completion of the interview, eligible respondents received a gift card.

### Data analysis

Each of the 15 interviews were assigned a participant ID and independently coded by two separate research team members trained in qualitative methods. The codes emerged from the interviews directly and thematic analysis was used. Coders met after analyzing the first interview to reconcile coding technique and resolve discrepancies. The interviews were coded sequentially in order of interview date until half of the interviews were completed. At this point, a preliminary codebook was developed between the two coding team members based on frequency and relevance of codes from these first 8 interviews. The subsequent 7 interviews were then coded in order of interview date, with the coding team members meeting weekly to update and edit the codebook with new and emerging themes and assess inter-coder agreement. After 15 interviews, all transcripts were reviewed by the coding team and assessed for recurring ideas. Codes were quantified and compared both within and between the three interview groups to determine that thematic saturation was reached. All interviews were eventually dual-coded, with all differences reconciled by consensus. All codes with corresponding interview passages were compiled and organized using NVivo software 12.0 (QSR International Pty Ltd, 2018).

The codebook was mapped onto the original conceptual model, the Anderson-Gelberg model adapted for vulnerable populations (Gelberg et al., 2000), incorporating predisposing factors (including traditional domains of participant demographics, marital status, employment and education, vulnerable domains of sexual orientation, homelessness, criminal history, mental illness and substance use) and enabling factors (including traditional domains of insurance, income and social support and vulnerable domains such as competing needs, transportation, telephone, case manager, public benefits, perceived barriers to care and ability to negotiate system). Study team members independently reviewed node reports and then discussed and assessed the findings as a group, iteratively identifying key themes from the interviews. In addition, a quantitative evaluation was performed for all codes by interview group (client/patient, medical, legal) in order to ascertain which topics were mentioned most frequently by which groups, and which themes emerge as common for all three groups. These quantitative results were triangulated with the research team discussions and organization of node reports, resulting in the identification of the final themes.

## Results

Demographic data for the 15 interviewees are presented in Table 2. Among the 15 participants, 8 were men and 7 were women; 8 identified as Black/African American and 7 identified as White. There were 6 participants in the patient/client group, 5 participants in the medical provider group, and 4 participants in the legal entities group.

**Table 2:** Participant demographics

	Patients/Clients	Medical	Legal
**N**	6	5	4
**Race/Ethnicity**			
**Non-Hispanic African American/Black**	3	3	1
**Non-Hispanic White**	3	2	3
**Gender**			
**Female**	0	4	3
**Male**	6	1	1
**Average Age (years)**	48.2	52	50

Three major themes emerged from the data: 1) Patients/clients highlighted the significance of psychosocial support and individual outlook more than medical and legal participants, who primarily focused on logistical factors such as finances, housing, and transportation; 2) Patients/clients expressed both medical and legal needs post-incarceration, though medical providers and participants from legal entities expressed concerns specific to their respective scopes of work; 3) A commonly shared perspective across participant groups emphasized the need for a low-barrier, collaborative, patient-centered approach to reentry with the goal of achieving self-sufficiency. Additional thematic quotations are provided in Table 3.

**Table 3:** Examples of quotations pertaining to the 3 thematic findings

1. Psychosocial vs. logistical barriers and facilitators1.1.1.	*“Without my family support and without X it would be pretty hard because you would have to navigate and try to find out who’s hiring, who’s gonna accept you, you know, and where can you get housing, where can you get food, where can you get clothing because, once you’re released, they just hand you a buncha paper and say, ‘Here.’” -Patient/client*
	“*I think if you have people like that, that are very, uh, focused on you, and helping you, it’ll really help you move along, and not be so depressed. Because getting out of the, uh, rehab facility and, and being incarcerated, it’s depressing. It’s extremely depressing, demoralizing, demotivating, and you know, and so, you get out of there, and you’re just like, ‘Oh, gosh, finally I can.’” - Patient/client*
	*“They’re in and out, in and out, because they have no stability. They don’t have the basics, so they do whatever they do to survive.” -Medical*
	*“I mean, there are so many, um, layers to it that you’ve got to figure out how to address it and how to get them to it, because it may not, it may be they want the help but they don’t know how to go about it. You, uh, “Yeah, I know it’s there. I don’t know how to get there, I don’t know how I’m going to afford getting there, and, in the meantime, I’ve got to find a place to live and I’ve got to eat,” so what do you do?...Yeah, these are common things. I mean, people need their basic needs met first.” - Legal*
2. Medical and legal entities’ competing priorities1.	*“They go to those services at the parole offices, but they’re handed, like, a sheet. And the sheet will have 12 different people on it to call. Well, these are services you might be interested in. They call 9 of them, the numbers don’t work. And, you know, if that’s what you’re supposed to get to help, and you already have these default position that nobody cares, and I’m given this by the TDCJ parole officer or case manager, I mean, I – I haven’t changed my way of thinking at all. In fact, it’s just cinched.” - Legal*
	*“I think a need that they don’t pay too much attention about is medical. Um, because I get their mentality. They’re trying to survive for today, you know?” -Legal*
3. Need for a low-barrier, collaborative, patient-centered approach with the goal of self-sufficiency1.1.1.1.	*“We’re enabling them to move forward in a manner that is gonna make them a productive citizen. They’re gonna contribute to society* versus *taking away from it, not only from a tax standpoint, but also, uh, having a family that works.” -Legal*
	*“I tell the client, you’re the advocate, you’re the one that needs to go in there and tell them. I can’t...I’m not gonna take over that role for you. So and that’s been my whole self-sufficiency process … you’ve gotta communicate that to the doctor.” - Legal*
	“*But you could always come back, you know. I’d rather it that way...So, they let you know they’re there. I call it like a partnership. It’s kind of, you know, a true trusted partner.” -Patient/client*
	*“‘Cause the great thing about these community people, and uh, these caseworkers is, you can call them, and talk to them, you know, tell ‘em what you’re going through, and that, that they’re, they’re kinda your buffer to hear, you know, what’s going on in your life. You know, when you come in for the appointment, you talk about anything. It’s very confidential, and you get to open up, and say, ‘Look, you know, I’ve, I’ve got this rash, or you know, had sex, and I didn’t use a condom,’ or, you can talk about anything.” -Patient/client*
	*“She’d follow up with me. ‘Hey, what are you doing now?’ I like that... they helped me out. I-I got what I needed, you know. But everything was there, you know, if you needed it … you feel like they care. Even the follow up, I’ll still get e-mails now. ‘[Redacted], what’s, you know, how’s everything going?’ It’s pretty cool...you never know what’s going to happen … But you could always come back.”-Patient/client*

### Theme 1: psychosocial vs. logistical barriers and facilitators

#### Social support and positive attitudes

Patients/clients spoke about barriers and facilitators related to psychosocial factors that affect their self-esteem and impact their experiences upon reentry. Multiple patient/clients stressed the need for support from family and community members to sustain positive reentry efforts:

*“That's one of the biggest things that people need to realize is when they come out of either incarceration or rehab facility, they need to have somewhere to go. They need to have family support, not just a, a shelter, but they need to have somebody where, that really cares about them, that watches over them, and wants them to do better, encourages them to do better.” -Patient/client*

Some patients/clients touched on social support as external motivation to do well. Knowing that someone else cared about them facilitated their own efforts for success upon returning to the community following incarceration:

*“I had somebody who cared and so, it made me care.” -Patient/client*

*“I was scared of navigating someplace that I have never been, you know, in 14 years and 8 months, I was scared of failure, you know? I didn’t wanna let a lotta people down, you know?” -Patient/client*

Patients/clients also emphasized the importance of a positive outlook, trust from others, and self-confidence as facilitators to reentry:

*“You know, you believe that lie that, you know, hey, you know, your life is never going to be the same. But this helped me get back on track with like that positive attitude. Hey, we're willing to help you, you know. We're going – we have relationships with people that will give you a shot, give you a chance.” -Patient/client*

*“When I got out in 2010, everybody saw that I was serious so, you know, and it feel good to where I could go to my sister house and she wouldn't watch me and I could tell the difference, you know, and I could get a car, you know, and I could borrow money and wouldn't have to ask for it twice. You know, it got the confidence back and it feel good. It's a difference, you know, when you're doing right and you're doing wrong.” - Patient/client*

In addition to the benefits of social support, patient/clients mentioned the negative consequences of attempting reentry without it:

“*‘Cause there’s a lot of people down there that feel like they just don't have anybody. You know, they're just down there, wasting away, and nobody cares about me, my life is over, and I don't know what to do, and you know, they kinda give up.” -Patient/client*

*“Yeah, just give someone, you know, some hope … Once you lose that, you're done. So, have that through a community or somebody who has been through it, I think it helps a little bit.” - Patient/client*

*“But the baseline of all this conversation basically is getting the best help support that you can, and that's people that really love you and care about you. You know, and if you don't have anybody, those are the people that rebound and go back to what their old habits were.” -Patient/client*

#### Basic needs

Participants from the medical and legal entities, conversely, noted mostly structural factors and logistical needs relating to employment, education, housing, and transportation as key determinants of successful reentry:

*“If the client gets their basic needs met – food, housing, clothes – then they're more … they usually will follow through, have a better follow-through if they get their basic needs met.” -Medical*

*“They're homeless, there is no job, … there are literacy issues, and there is paperwork that you have to fill out to... get into care. Then, if you're in special programs, there are probation fees. There is a certain time you have to be back, so I can't go to [the HIV clinic] and sit hours and hours and hours. I don't have the money to catch the bus. So, I just think they just don't have the support and the means to be successful, most of them.”* -*Medical*

Participants representing legal entities mirrored the perspectives of the medical providers:

*“ … if they don't have anywhere to lay their head or a shelter, nothing else matters.” -Legal*

*“And, uh, employment because, again, if they don't have a job to feed themselves or get housing, they're gonna do whatever it takes to survive which is possibly to reoffend. And we don't want that.” -Legal*

*“We know that when they have a good paying job with good benefits, um, and good transportation and good housing, their chances of going back are slim.” - Legal*

### Theme 2: medical and legal entities’ competing priorities

#### Medical providers: stigma, documentation, and sustained treatment engagement

Medical providers perceived issues related to their field as main barriers to successful reentry and medication adherence:

*“So, that's the biggest challenge, is to get them well here and then for them to be dumped back into a system and not be linked to care, and still go through the issues that they go through with trying to be accountable and stay up on their medication.” - Medical*

“*Getting their medication...I think that's a big challenge. And, uh, getting the necessary documents to get back into care.” -Medical*

One provider pointed to issues surrounding an HIV diagnosis and health literacy as foundational barriers affecting reentry:

*“The patient can understand the HIV, but, if the people around them don't understand HIV and continue to treat them like a disease or, you know, as contagious...I've heard a mom break down because, ‘Oh, I can't kiss my babies anymore.’ Yes, you can. ‘Oh, my babies can't eat after me.’ So, she's feeling like she's going to lose her closeness or her bonding or her cuddling with her child because of lack of education...I can teach my patient all day, but if the people that they need the most – ... That's a scary place to be. Even though the medications have gotten better, that's still scary.” - Medical*

Some providers pointed to patient documentation issues and the time-consuming nature of staying current with medications:

*“Well, you got to get a XXX [state] license because it's so hard to navigate the system without a current ID with a current address...And to me it seems really simple, picture ID, proof of address, proof of income, but for some people that's just – they can't seem to get that together.” -Medical*

*“I say, ‘When you get down to ten pills in your bottle you – your next thought is gonna be where am I gonna get my next month's supply? Do I have a refill? Do I know the pharmacy? Am I updated with my program?’...it's like a full-time job for the patient” - Medical*

One alternative medical perspective touched on the phenomenon of patients not addressing medical concerns and medication refills because they do not necessarily feel sick and have other things they choose to prioritize:

*“Sometimes they just, ‘I'm doing fine, I'm feeling fine. I just don't want to deal with that now.’” -Medical*

#### Legal barriers: returning to the community with a criminal record

Participants from legal entities focused on barriers to reentry that relate to their own field, including navigating life with a history of incarceration or conviction(s), in addition to probation and parole requirements:

*“That's the problem with our society to begin with...that's why it's so hard for people coming out of prison to get jobs, because nobody forgives them for a mistake that they made. You know, you steal something, well, no, um, salesperson, or sales, wants you to work there, ‘Sorry. You steal.’” - Legal*

“*People don't realize that some nursing homes and assisted living facilities, you can't have a criminal history living there.” - Legal*

“*But the ones who are on parole, I already have to go to my parole officer. I have to go see him. I gotta go report to these meetings I really don't wanna attend for a year or so. They're tired of going up there. So if you wanna say, let's go see our reentry coordinator – let's go see my reentry case manager, it's like, uh, no, I'd rather be out here hitting my head against the wall.” - Legal*

When asked which clients face the most challenges upon reentry, one legal participant stated:

*“All of ‘em...It depends on the charge, honestly. Um, if they're aggravated charges, they have a – just as much difficulty as a sexual offense charge because aggravated charges are still charges. They're a liability to anyone who hires them or even going to school … so they... have the same level of difficulty finding work, housing … education … There still will be doors shut.” - Legal*

When asked what clients need the most assistance with, the same legal participant pointed to length of incarceration as a key determinant:

*“Depends on … how long they were locked up. It would be education on the public transit system. Um, of course, employment. Um, and just simple, everyday skills. How to dress for an interview. Um, how to communicate between your employer and even your co-worker.” - Legal*

One legal participant cited averting legal authority as a potential barrier to fully engaging in services upon reentry:

*“If they've got a lot of criminal stuff going on, they're not going to give you truthful information.” - Legal*

### Theme 3: need for a low-barrier, collaborative, patient-centered approach with the goal of self-sufficiency

Patients/clients, medical providers, and participants from legal entities shared an emphasis on the need for an accessible, collaborative, patient-centered approach that helped patients/clients achieve autonomy. In discussion about who bears responsibility for successful connections to care in the community, one medical provider pointed to a collaboration between patients/clients and their providers:

*“I do think, ultimately, it is the responsibility of the person who has the illness, um, however, um, in healthcare, if we're gonna be successful in helping people, we have to recognize that we're a big part of helping to enable that person to be able to take responsibility … So we have a responsibility to help create a sense of responsibility, I think, in the person that we're treating. And I think the best healthcare professionals are good at that.” - Medical*

Another medical provider discussed the team nature of the patient-provider relationship:

*“ … and my thing is I tell them we're on the same team. So, it's a partnership between the client and me, you know, being an advocate for them.” -Medical*

One medical provider praised the inclusion of community health workers as part of the care team, sharing they can connect well with patients and personalize care:

*“I've got … a lot of belief in the idea of community health workers. People who the patient believes that they can relate to because they've, sort of, walked the same, kind of, walk. … It seems like it would be a very effective way. As opposed to the doctor saying, you've gotta go do this, and moving on to the next patient, I think it's a warm connection.”-Medical*

In agreement with medical providers, patient/clients underscored the importance of working together with community health workers and other service providers and the impact that such collaboration and partnership can have on their attitude and reentry experiences:

*“If [a community health worker] actually coming down to visit me and talk to me about trying to help me along, and get me the medication, and get me the resources that I need, I think that would be huge. I think that would change somebody's mentality, change their outlook, change, change the way they feel.” -Patient/client*

*“I mean, I don't feel like I'm just some number. I feel like, you know, she really is truly trying to figure out a way to help me. So, that's always a good thing.” - Patient/client*

Though most patients/clients did not have prior experience with a community health worker, they praised the concept of a community health worker that has experiences in common with the people whom they serve – an added credibility that allows for effective collaboration and encouragement upon reentry:

*“‘Cause you can’t take advice from somebody that can’t relate to you.” -Patient/client*

*“Yeah, that's good because they know. They know the dos and don'ts, you know, the whats and whatnot. They can really reach out and help somebody because they know where they come from.”-Patient/client*

*“That person’s … trying to stop you from going through what he went through, trying to help you not go have the problem that he had. I think that's excellent … And a person will work with them, too, ‘cause they'll know that this guy done been through this. I'm listening, you know what I'm saying? I'm gonna pay attention to what he's telling me and do what he's telling me to do. Yeah. ‘Cause he's been through it.”-Patient/client*

Participants from legal entities, like those from medical and patient/client groups, condoned the use of community health workers as a means of reentry support:

*“A lot of ‘em didn't have the social skills or the know, know-all to – or how to communicate with someone if they're needing help or assistance. Some of them may be intimidated by authority figures. So I think that advocate would be great.” – Legal*

*“Some people may be too timid or afraid to go out there by themselves. And to have someone say, “Hey, it's all right. Let me show you the way,” one or two times – it would give them that self-esteem and that determination. And that will to say, “I can do this.” And go do it.” -Legal*

One legal participant echoed the sentiments of the patient/client group, explaining that a community health worker with shared lived experience to those they serve is key:

*“I think hearing from someone who has been in their shoes makes a big difference … And it's, I need somebody who looks like me and has been where I've been and sounds like me, to tell me. And then I'm gonna do it. And I think that's gonna make the difference. I really do. I think it's gonna help a ton.” - Legal*

One participant from the legal entity succinctly summed up their patient-centered approach, stressing the need to meet patient/clients where they are:

*“Success is measured based on that person's goal, not mine.”- Legal*

Similarly, a medical participant stressed the importance of individualized, patient-centered health care, particularly related to substance use:

*“I'm gonna keep nudging you to try to … get you off the meth or crack cocaine, but if you're not ready yet, I'm not gonna push you ‘cause … that's not gonna help you.” -Medical*

Participants from the legal entities, also emphasized the need for eventual patient/client independence:

*“It's not on us to make it work. We'll be there to do whatever for them, but at the end of the day, it's on them to make it work. And so we impress upon them from the get-go that this is a, you know, a hand up, not a hand out. They have to work the program, and if they're not ready, we'll be there when they are” - Legal*

*“The mission is to get the clients to self-sufficiency so they do not go back. To reduce recidivism rate in our community.” -Legal*

*“It's, uh, it's almost like looking at your children. You know, we've got to let go at some point, and I know we care about these and we know what's going to happen, and it's just going to be a vicious cycle, but we have to learn to let go.” - Legal*

In line with the legal participants, patients/clients also stressed the value of self-sufficiency:

*“You can’t take the freedom for granted, you know, to have your own checking account, have your own credit cards, to be able to take your own key and open your door, get in your car and go eat what you wanna eat, you know? -Patient/client*

*“You know me working, me paying my own bills. Me being able to feed myself, me being able to pick up the phone, ‘Hey, ma, you need anything?’ You know now I’m feeling more confident about myself and, hey, I’m all right.” - Patient/client*

## Discussion

Much of the literature outlining the challenges of reentry following incarceration is limited to the perspective of those released, often excluding the viewpoints of community medical, legal, and social service providers who interact with this population. In the current study, we obtained data from a unique group of stakeholders - medical providers and administrative staff from jail-based clinics, representatives from the Sheriff’s office, staff from a community HIV clinic that serves justice-involved individuals, leadership and staff from acommunity reentry program focused on legal advocacy, and formerly incarcerated patients/clients of these entities - and found key similarities in their perspectives on reentry. Among the three groups, participants acknowledged the significance of logistical barriers to community reentry and obtaining basic needs, benefits of team-based and patient-centered approaches to reentry, and an end goal of self-sufficiency. However, there were key differences between groups in additional themes viewed as relevant. Patients/clients focused heavily on psychosocial barriers and facilitators to reentry, while the other groups chiefly emphasized logistical needs. Additionally, specialty-specific focus seen in the medical and legal groups highlighted the siloed services and challenges of competing priorities that patients/clients returning from incarceration experience. Data gleaned from the qualitative interviews were intended to assess feasibility and inform implementation of a community health worker reentry intervention for these patients/clients aimed at enhancing HIV and substance use disorder health outcomes and reducing recidivism.

Participants from medical and legal entities primarily noted logistical barriers (housing, transportation, documentation, employment) whereas the patient/client group emphasized that social support and encouragement from people close to them were just as important as other practical facets of reentry. The latter group discussed wanting to succeed because they knew people close to them were invested in and hoping for positive outcomes. Further, they noted how it built their self-confidence when family members who previously did not trust them started to do so and how that made them want to continue to do even better. These sentiments mirror those presented in Rozanova et al. (2015), who found that positive social relationships with family members and health care providers enhanced ART adherence in formerly incarcerated patients, as well as Dong et al. (2021), whose qualitative work underscores the critical value of patient-provider relationships in HIV care during the reentry period. Patients/clients also discussed the negative sides of lacking social support: giving up and returning to “old habits” that may have contributed to poor health and legal outcomes in the first place. They spoke of overcoming feelings of hopelessness, vulnerability, and mistrust of society as being a crucial piece of dealing with the logistical barriers of reentry. This is consistent with the perspectives of patients living with HIV in Taylor et al. (2018), who noted lack of social support as a major barrier to attending HIV-related medical visits. These results are also in line with those of Denney et al. (2014) in which formerly incarcerated participants, though deemed successful by their reentry programs, described lacking social support as a major struggle upon release from incarceration.

Narratives from all three participant groups in this study support the utilization of community health workers, often seen in interventions like the Transitions Clinic model (Shavit et al., 2017), to assist patients/clients in navigating reentry. Community health workers provide an array of education, advocacy, and systems navigation services that can lessen the common barriers experienced by the justice-involved population. Aminawung et al. (2021) found that over a third of formerly incarcerated patients who met with a community health worker during the reentry period received assistance with social determinants of health such as housing, health insurance, transportation, and government benefits, addressing the basic needs highlighted by the medical and legal participants in the current study. Community health workers, particularly those who have similar life experiences as the patients/clients they are serving, may also contribute to the much-needed psychosocial support in the transition back to the community post-incarceration. In the absence of family or friends, many people coming out of jail and prison and living with HIV have found their greatest source of psychosocial support comes from their HIV care team (Rozanova et al., 2015). As part of a care team, community health workers stand as a solution to both the logistical barriers stressed by medical and legal entities and the psychosocial aspects emphasized by the patients/clients.

Medical providers and representatives from legal entities that serve formerly incarcerated individuals concur that people reentering the community following incarceration face myriad logistical barriers to health and stability. However, participants from each group primarily discussed issues with which they are most familiar and perceived barriers related to their own respective fields of work as paramount, reflecting the competing priorities with which the reentering population contends. Medical providers spoke about medication adherence and HIV stigma, seldom mentioning the obstacles of parole or probation requirements and other legal burdens that patients carry, results that are consistent with Sidibe et al. (2015). Participants from legal entities focused on barriers related to navigating the community (such as finding housing and employment) with a criminal record and the challenges that this experience poses. This siloed approach and incomplete awareness of each group’s goals and services pose an additional burden that ultimately falls on patients/clients, likely contributing to recidivism. Previous reentry interventions among PWH have included intensified case management or linkage coordinators (Rich et al., 2001; Wohl et al., 2011; Zaller et al., 2008) but have not directly engaged other sectors in the community– such as the housing system, food banks, employers who hire formerly incarcerated individuals, education centers, religious entities, mental healthcare, substance use treatment, correctional supervision. HIV case management services are typically clinic-based, rely on passive referrals to outside agencies and require individuals to access care in the clinic prior to accessing social services. The results of the current study complement those found in Dong et al. (2021) whose multi-stakeholder exploration of HIV and reentry yielded multiple solution ideas to interruptions along the HIV care cascade for justice-involved patients.

Our results point to the need for integrated, low-barrier reentry interventions that serve as a “one-stop shop” for reentering patients/clients living with HIV and a history of substance use to address health, employment, housing, and other legal and logistical needs. Such interventions, often referred to as medical-legal partnerships, exist across the United States and serve varying populations such as cancer patients, families with children, and veterans. In New York and Connecticut, for instance, medical-legal partnerships that had legal professionals embedded in hospitals showed positive physical and mental health and legal outcomes for veterans (Tsai et al., 2017). In Palo Alto, CA, adding an on-site lawyer in medical settings facilitated increased access and uptake of social services in families with children (Weintraub et al., 2010). Few medical-legal partnerships, however, have been tailored to meet the needs of people returning to the community after incarceration. One such partnership exists in New Haven, CT, and situates lawyers and law students in the clinic so patients can access services such as legal screenings, help with child support and custody, and information about food stamps and other cash assistance without travelling to multiple locations (Benfer et al., 2018); however, the outcomes of this partnership have not yet been evaluated. Given the results of the current study, coupled with rates of HIV care reengagement after release from incarceration as low as 36% (Ammon et al., 2018; Iroh et al., 2015) and relatively high rates of recidivism in this population (Fu et al., 2013; Marlow et al., 2008), more comprehensive interventions that position medical personnel, legal advocates, and other reentry service providers as partners can reduce barriers to care and improve reentry and health outcomes.

Lastly, study findings indicate the need for a patient-centered collaboration between all three stakeholder groups that encourages patient/client and medical and legal service provider input and communication. Like participants in Rozanova et al.’s (2015) qualitative work, patients/clients in the current study expressed the desire to feel part of a team, not “just some number,” a notion that could encourage continued engagement in care. The concepts of partnership and teamwork are also consistent with Taylor et al.’s (2018) finding that patients and providers believe shared decision-making to be a vital driver of patient engagement in HIV care. Patients/clients in the current study also valued teamwork in relation to continued commitment to nonmedical facets of reentry like employment. One patient/client participant described a positive feeling about getting check-in emails from his reentry program even after obtaining employment. Sensing the partnership and exchange of information was considered helpful; knowing he could always communicate with his reentry team helped sustain confidence, a key driver of self-sufficiency, which was a goal noted by all three participant groups. This shared goal further supports the need to harmonize medical and legal reentry services and utilize trained peer support such as community health workers to reduce gaps in care, minimize disengagement from services, and increase accountability.

The current study is not without limitations. First, patients/clients interviewed in the community were already linked with HIV and/or reentry services. Additional inquiry is required to examine the viewpoints of those not yet received into such supportive environments. Additionally, the purposive sample was diverse with respect to the roles that people had rather than their race, gender, and ethnicity. Though roughly half of interviewees were female, all patients/clients identified as male, reflecting the male majority seen in the local jail population and justice-involved settings at large. Capturing data from a more racially and ethnically diverse group that includes justice-involved women may help create a more complete picture of the reentry experience for people living with HIV and substance use disorder and inform a richer intervention. Despite these limitations, our findings suggest a clear necessity for collaborative, multi-sectoral, co-located service provision that addresses psychosocial and logistical needs.

## Conclusion

This study aimed to highlight perspectives of a variety of stakeholders - individuals recently released from incarceration living with HIV and SUD and the medical and legal providers who serve them - in hopes of informing a community health worker reentry intervention. The results of this qualitative study support and extend existing literature documenting the barriers and facilitators to successful reentry. Our findings underscore the notion that a successful reentry intervention includes an individualized, longitudinal approach which incorporates psychosocial needs, involves a medical-legal partnership, and focuses on establishing self-sufficiency.

## Data Availability

The data generated during and/or analysed during the current study are not publicly available due to protecting the privacy and confidentiality of the interviewees but are available from the corresponding author on reasonable request.
