# Correction to: Patient, medical and legal perspectives on reentry: the need for a low-barrier, collaborative, patient-centered approach

**DOI:** 10.1186/s40352-022-00179-5

**Published:** 2022-04-30

**Authors:** Zoe Pulitzer, Maria Box, Laura Hansen, Yordanos M. Tiruneh, Ank E. Nijhawan

**Affiliations:** 1 Department of Internal Medicine, Division of Infectious Diseases and Geographic Medicine, University of Texas Southwestern Medical Center, 5323 Harry Hines Blvd., Dallas, TX 75390 USA; 2 Department of Emergency Medicine, University of Texas Southwestern Medical Center, 5323 Harry Hines Blvd., Dallas, TX 75390 USA; 3 University of Texas Health Sciences Center at Tyler, 11937 US-271, Tyler, TX 75708-3154 USA; 4 Parkland Health and Hospital Systems, Correctional Health, Dallas, TX 75235 USA

**Correction to: Health Justice 9, 37 (2021)**.

10.1186/s40352-021-00161-7

Following the publication of the original article (Pulitzer et al., 2021) the authors noticed that the published version of the manuscript has blinded citations.

The original article (Pulitzer et al., 2021) has been updated.
